# Murine colon organoids as a novel model to study *Trypanosoma cruzi* infection and interactions with the intestinal epithelium

**DOI:** 10.3389/fcimb.2023.1082524

**Published:** 2023-03-09

**Authors:** Hellen Daghero, Romina Pagotto, Cristina Quiroga, Andrea Medeiros, Marcelo A. Comini, Mariela Bollati-Fogolín

**Affiliations:** ^1^ Cell Biology Unit, Institut Pasteur Montevideo, Montevideo, Uruguay; ^2^ Redox Biology of Trypanosomes Lab, Institut Pasteur de Montevideo, Montevideo, Uruguay; ^3^ Department of Biochemistry, Faculty of Medicine, University of the Republic, Montevideo, Uruguay

**Keywords:** Chagas disease, HT-29 cells, intestinal organoids, murine colon organoids, *Trypanosoma cruzi*

## Abstract

Chagas disease (CD) is a life-threatening illness caused by the parasite *Trypanosoma cruzi* (*T. cruzi*). With around seven million people infected worldwide and over 50,000 deaths per year, CD is a major public health issue in Latin America. The main route of transmission to humans is through a triatomine bug (vector-borne), but congenital and oral transmission have also been reported. The acute phase of CD presents mild symptoms but may develop into a long-lasting chronic illness, characterized by severely impaired cardiac, digestive, and neurological functions. The intestinal tissue appears to have a key role during oral transmission and chronic infection of CD. In this immune-privileged reservoir, dormant/quiescent parasites have been suggested to contribute to disease persistence, infection relapse, and treatment failure. However, the interaction between the intestinal epithelium and *T. cruzi* has not been examined in depth, in part, due to the lack of *in vitro* models that approximate to the biological and structural complexity of this tissue. Therefore, to understand the role played by the intestinal tissue during transmission and chronic infection, physiological models resembling the organ complexity are needed. Here we addressed this issue by establishing and characterizing adult stem cell-derived colonoid infection models that are clinically relevant for CD. 3D and 2D systems of murine intestinal organoids infected with *T. cruzi* Dm28c (a highly virulent strain associated with oral outbreaks) were analyzed at different time points by confocal microscopy. *T. cruzi* was able to invade and replicate in intestinal epithelial primary cells grown as intact organoids (3D) and monolayers (2D). The permissiveness to pathogen infection differed markedly between organoids and cell lines (primate and intestinal human cell lines). So far, this represents the first evidence of the potential that these cellular systems offer for the study of host-pathogen interactions and the discovery of effective anti-chagasic drugs.

## 1 Introduction

Chagas disease (CD) is a zoonotic disease, endemic in Latin America, caused by the protozoan parasite *Trypanosoma cruzi* (*T. cruzi*) (Rassi et al., 2010). The parasite is mainly transmitted to humans and other animals by hematophagous insects from the Triatominae subfamily. However, congenital, oral, and blood-borne transmission has also been reported for CD (Pereira et al., 2009; Requena-Méndez et al., 2015).

*T. cruzi* presents a complex life cycle that involves a transition from replicative to non-replicative but highly infective stages. In the insect’s gut, epimastigote forms multiply and differentiate into infective and non-replicative metacyclic trypomastigotes (MTs). The trypomastigotes are able to infect a wide range of mammalian cell types. In the cytosol of the host cell, the trypomastigotes differentiate into amastigotes, which upon several cycles of binary division, transform into motile trypomastigotes that lyse the cell mechanically. In most cases, the acute phase of CD presents mild and non-specific symptoms and is characterized by high levels of extracellular parasites. In about 30 - 40% of infected people, the disease develops to a chronic stage characterized by parasite colonization of several tissues such as the heart, colon, and gut mesenteric ganglia and muscle (Lidani et al., 2019). Disease morbidity and mortality are associated with cardiac and/or digestive damage and dysfunction. However, tissue tropism and clinical manifestations depend on many factors, especially the parasite genetics and the immune response of the host, making it difficult to predict the clinical development of the disease (Andersson et al., 2003). To date, only two drugs are available for CD treatment: benznidazole and nifurtimox. Both have been demonstrated to be effective in the acute phase, but they present considerable side effects and are not useful for treating the chronic CD stage.

The host’s gastrointestinal tract is a key site for *T. cruzi* persistence since dormant/quiescent parasites residing in this immune-privileged location have been suggested to contribute to disease persistence, infection relapse and treatment failure (Ward et al., 2020). Parasite nests have been frequently detected in the muscle layer surrounding the colon of chronically infected mice. However, little is known about the capability of *T. cruzi* to establish infections and/or damage the intestinal epithelium. Moreover, murine models of CD are not only disputed because of ethical concerns but also because they have many limitations to model chronic CD (Fonseca-Berzal et al., 2018). Some of these limitations can be overcome by *in vitro* cell culture methods (Martello et al., 2013; Rodríguez et al., 2020).

Recent advances in stem cell biology and 3D culture systems have led to the development of valuable tools for exploring host-parasite interaction *in vitro*. Intestinal organoids, consisting of a differentiated, polarized epithelium with a central lumen, can be obtained by culturing intestinal stem cells (ISCs) (Sato et al., 2009). The ISCs reside at the crypt base within specialized niches where they self-renew to maintain a functional epithelium throughout life (Barker, 2014). When cultured under appropriate conditions, the ISCs can divide and rearrange themselves recapitulating the architecture and function of the tissue. The different epithelial cell types are present in these cultures, both absorptive and secretory lineages, hence representing a valuable system for performing functional studies of the intestinal epithelium. These advances in intestinal stem cell-derived organoid culture have broadened the *in vitro* repertoire of studies for host-parasite interactions for a wide range of enteric parasites (Wilke et al., 2019; Holthaus et al., 2021; Holthaus et al., 2022; Lamisere et al., 2022).

Here we addressed the establishment of 2D and 3D murine colon organoids (colonoids) to investigate *T. cruzi* tissue infection. Our data show that *T. cruzi* is able to invade the epithelium from both sides of the organoids (apical and basolateral surface) and that invasion and replication appear to be cell-type specific.

## 2 Materials and methods

### 2.1 Mammalian cell culture

HT-29 (ATCC HTB-38) and Vero (ATCC CCL-81) cells were cultured in Dulbecco’s Modified Eagle Medium (DMEM) supplemented with 10% (v/v) Fetal Bovine Serum (FBS) in 25 or 75 cm^2^ tissue culture flasks. Cells were routinely incubated at 37°C, 5% CO_2_ in a humidified incubator and subcultured when reaching a 70-80% confluence for HT-29 or 40-60% for Vero cells.

### 2.2 Murine colon-derived organoid culture

Colon tissues of C57BL/6J mice (6-8 weeks-old male and female) were collected by dissection. Three cm long tissue sections were flushed with sterile PBS supplemented with 1% (w/v) penicillin/streptomycin. Samples were sliced longitudinally, cut into 0.5 cm fragments and then washed in cold PBS until the supernatant was clear. Following a 20 min incubation in ethylenediamine tetraacetic acid (EDTA) 10 mM in PBS with gentle agitation, fragments were resuspended in shaking buffer (0.1% (w/v) BSA in PBS) and pipetted up and down 5 times with a 10 mL pipette to facilitate crypt release. Upon examination by microscopy, crypt-containing fractions were pooled and filtered using a 70 μm cell strainer. After a centrifugation step at 200 g for 5 min at 4°C, the isolated crypts were counted and resuspended in Reduced Growth Factor Cultrex^®^ Basement Membrane Extract (R&D Systems) at 250 crypts/20 μL matrix. The crypt/matrix droplets were incubated for 10 min at 37°C to allow matrix polymerization and organoid medium was added (Advanced DMEM/F12, Gibco), 1% (w/v) L-glutamine, 1% (w/v) penicillin/streptomycin, 50% (v/v) L-WRN conditioned medium, 10 nM gastrin (PeproTech), and 10 μM Y-27632 (PeproTech). Organoids were incubated at 37°C in a 5% CO_2_ humidified atmosphere. For maintenance, the medium was renewed every 3 days with an organoid medium without Y-27632. Organoids were subcultured every 4-7 days in a ratio of 1:2 or 1:3 and used at day 3 for 3D infection assays.

### 2.3 Murine organoid-derived monolayer

For growing intestinal organoids in a monolayer format, a black clear-bottom 96-multiwell plate (Corning) was pre-coated with 50 μL per well of a 1:10 Cultrex-BME^®^-PBS solution. The plate was incubated for 2 h at room temperature and then at 37°C until use. Full-grown 3D organoids were detached from matrix domes using ice-cold PBS and then centrifuged at 300 g for 5 min at 4°C. The cell pellet was resuspended in TrypLE Express 1X (Gibco) supplemented with 10 μM Y-27632 and incubated at 37 °C for 5-7 min. For facilitating cell disaggregation, the suspension was vortexed every 2 min. After TrypLE inactivation with complete culture medium, the cells were counted and seeded in the pre-coated plate (5x10^4^ cells/well). The culture medium was supplemented with 2.5 µM CHIR99021 (PeproTech), 10 µM SB202190 (Sigma-Aldrich), 1 mM N-acetyl cysteine (Sigma-Aldrich) and 10 mM Nicotinamide (Sigma-Aldrich), and replaced every 2-3 days.

### 2.4 Generation of infective *Trypanosoma cruzi*

Epimastigotes of *T. cruzi* strain DM28c (clone TcI) were cultivated in batch for 12-15 days in liver infusion tryptose (LIT) medium supplemented with 10% (v/v) FBS, 100 U/mL of penicillin, 100 μg/mL streptomycin and 40 µM of Hemin at 28°C. Next, Vero cells (2.5 x 10^5^ cells/mL in T-25 flasks containing DMEM + 10% (v/v) FBS) were infected at a 10:1 (parasite:Vero cell) ratio with MTs present in the supernatant of the aged epimastigote´s culture. The culture was monitored for the proliferation of intracellular parasites and the medium (DMEM + 2% (v/v) FBS) was replaced every 48 h. When high amounts of extracellular trypomastigotes were detected (6-7 days post-infection), the supernatant was collected, centrifuged at 1250 g for 10 min and the parasite pellet resuspended in fresh DMEM + 10% (v/v) FBS. After 4-24 h incubation at 37°C and 5% CO_2_, swimming parasites were recovered from the supernatant by centrifugation at 1250 g for 10 min, resuspended in complete organoid culture medium and counted under the light microscope. The parasite density was adjusted by dilution in the complete organoid culture medium. The procedure was performed immediately before the organoid´s infection assay.

### 2.5 Infection assays

For the 3D infection assays, each independent experiment was performed with organoids obtained from different mice (4 different organoid lines), and from both sexes. Organoids were removed from the BME matrix domes, washed with PBS and centrifuged at 300 g for 5 min at 4°C. To adjust the number of parasites to the corresponding MOI to be tested, organoids contained in a single well of a 6-well plate were dissociated into a single-cell suspension by incubating them with TrypLE for 5 min at 37°C and used as a proxy to determine the number of viable cells per well (counted under a light microscope). Once the number of cells was determined, the number of parasites needed was calculated considering the desired MOI. Then, intact organoids were removed from the BME matrix domes, washed with PBS and centrifuged at 300 g for 5 min at 4°C. Upon removal of the supernatant, the organoid pellet was incubated for 2 h at 37°C with the appropriate number of infective *T. cruzi* trypomastigotes in 100 μL of complete organoid medium supplemented with Y-27632. Thereafter, the infected organoids were centrifuged at 300 g for 5 min, resuspended in BME matrix, plated in a black clear-bottom 96 multiwell plate in 7 μL drops and cultured for 72 h in complete organoid medium supplemented with Y-27632.

For the infection of organoids in 2D, each independent experiment was performed with organoids obtained from different mice (2 different organoid lines) and from both sexes. The wells for the organoid monolayer were pre-coated with a BME matrix 1:10 dilution in PBS for 2 h at 37° C. The single-cell suspension from organoids was obtained by incubating the suspended organoids with TrypLE for 5 min at 37°C and the MOI was adjusted using the number of cells seeded per well. For Vero and HT-29 cells was 1.5x10^4^ cells/well and for organoid single-cell suspension was 5x10^4^ cells/well. Once the monolayers were established, the infective trypomastigotes were added directly to the cell culture and incubated for 2 or 24 h. Then, the medium was removed and the monolayer was washed thrice with PBS (pre-warmed at 37°C). Complete organoid medium supplemented with Y-27632 was added and the culture plate was further incubated for 48 h to 72 h.

### 2.6 Immunofluorescence staining

Infected organoids were washed with PBS three times and fixed with 4% (w/v) paraformaldehyde (PFA) in PBS for 1 h at room temperature (RT). Permeabilization was performed by treating the samples with 0.5% (v/v) Triton X-100 in PBS for 15 min at RT. After blocking with 2% (w/v) BSA in PBS for 2 h at RT, the samples were incubated with parasite-specific primary antibodies (polyclonal rabbit serum anti-*T. cruzi* trypanothione reductase or polyclonal mouse anti-*T. cruzi* mitochondrial peroxiredoxin), or anti-Ki67 antibody (ab 15580 from Abcam), Wheat Germ Agglutinin (WGA) Alexa Fluor 555 (Invitrogen 1:1000 dilution) and methyl green (4 μg/mL) or Hoechst 33342 (1:1000 dilution) in 2% (w/v) BSA, 0.1% (v/v) Triton X-100 in PBS overnight at 4°C. Next, the samples were washed three times with PBS and incubated with the secondary antibody anti-rabbit IgG Alexa Fluor 488 (Invitrogen) (1:500 dilution), or anti-rabbit IgG Cy5 (Invitrogen) (1:500 dilution), anti-mouse IgG Alexa Fluor 488 (1:300 dilution) and Phalloidin Texas Red (ThermoFisher) (1:100 dilution) or Phalloidin-Alexa Fluor 647 (ThermoFisher) (1:200 dilution) for 1 h in the dark at RT.

### 2.7 Image acquisition and analysis

Confocal images were acquired using Zen Black software (Zeiss; Darmstadt, Germany) on a laser confocal microscope Zeiss LSM 880 equipped with 25X (Gly immersion) and/or 40X (oil immersion) objectives and 488 nm, 561 nm and 631 nm lasers. Images were processed using Fiji software (Schindelin et al., 2012). For the detection of parasites in infected 3D-organoids, Z-stack images were obtained from 5 random fields per condition and quantification was performed with the 3D Object counter plugin from Fiji, using nuclei staining for counting all cells and trypanothione reductase or mitochondrial peroxiredoxin signal for counting *T. cruzi* amastigotes.

### 2.8 Statistical analysis

Data were expressed as the median and interquartile range of replicates from one representative experiment, out of three independent experiments executed, unless otherwise indicated. The statistical analysis was performed using GraphPad Prism. For comparing two groups, an unpaired Mann-Whitney Test was applied. A p-value of less than 0.05 was considered significantly different.

## 3 Results

### 3.1 *Trypanosoma cruzi* is able to invade colon-derived organoids from the basolateral side

**Figure 1:**
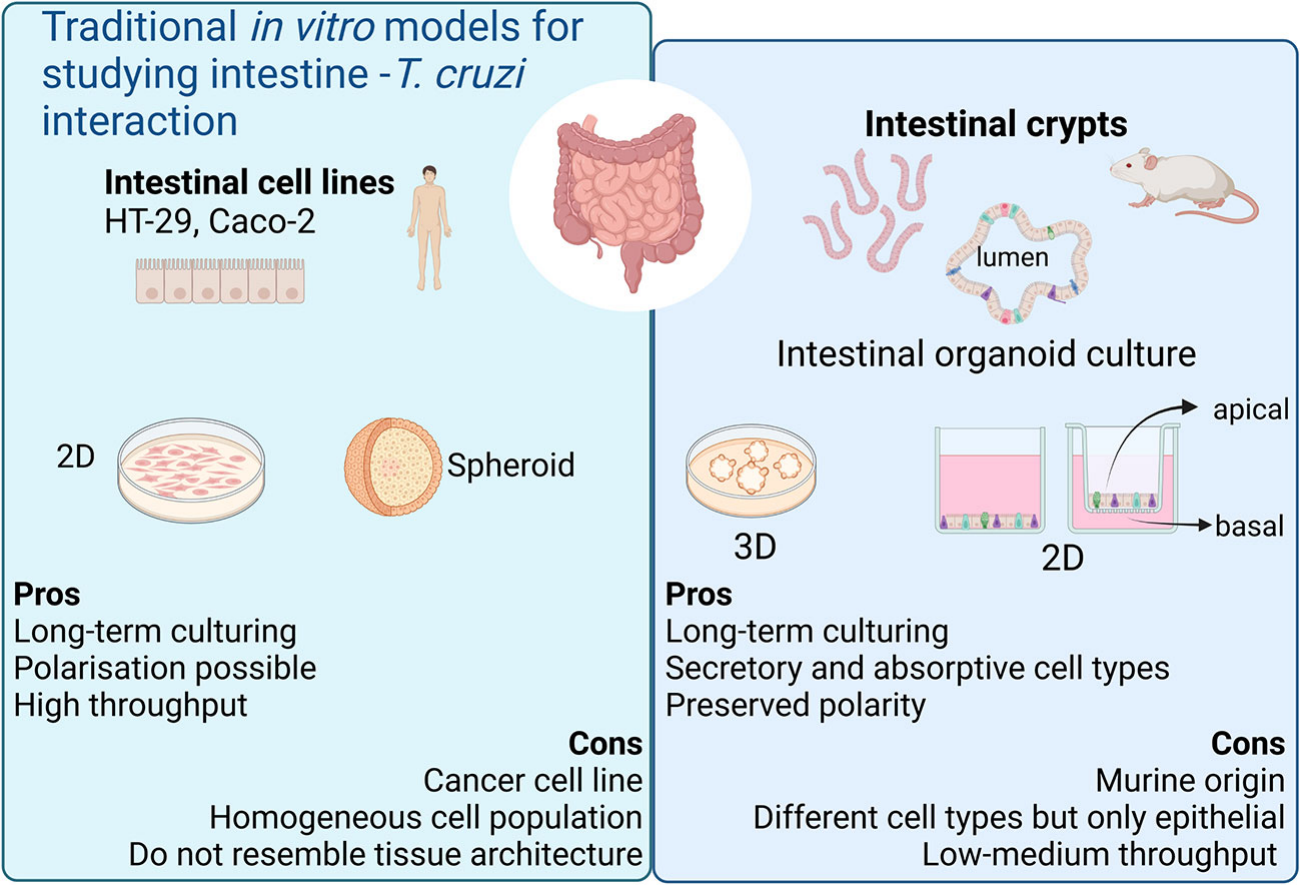
Current *in vitro* models for modeling chronic Chagas Disease. Created with BioRender.com (agreement number BV2506JOHI).

**Figure 2:**
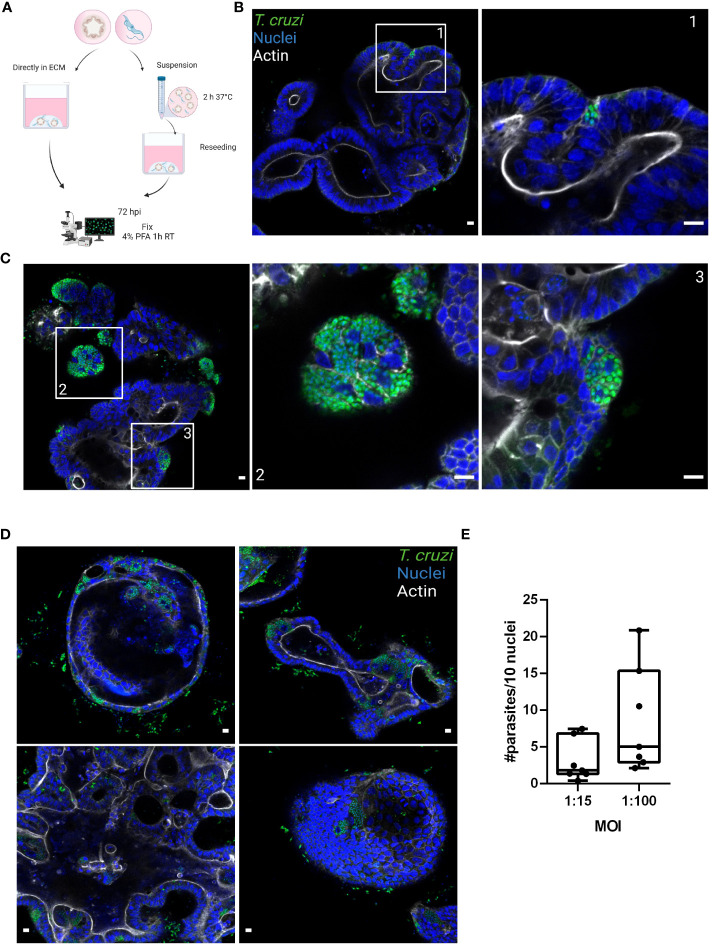
Basolateral infection of organoids by *T. cruzi*. **(A)** Schematic representation of the infection protocol. Murine colon-organoids were removed from the matrix and either directly re-plated with the parasites or incubated for 2 h in suspension at 37°C before being re-plated. At 72 h post-infection parasite load was assessed by confocal microscopy. Created with BioRender.com (agreement number RW2506JDYE) **(B)** Representative image of organoids infected with parasites (MOI 1:15) included directly in the matrix. **(C)** Representative image of organoids infected with parasites in the absence of matrix. **(D)** Organoids infected with a MOI of 1:100 show discrete parasite nests. **(E)** Number of intracellular parasites every 10 cells over a 72 h period of infection. Data from one representative experiment out of 2 independent experiments (different organoid lines per experiment). Data are expressed as median and interquartile range and compared using Mann-Whitney test (n = 7). Scale bar: 10 µm. Nuclei (blue): methyl green staining, *T. cruzi* (green): anti-trypanothione reductase immune-staining, and Actin (white): phalloidin Texas Red staining.

**Figure 3:**
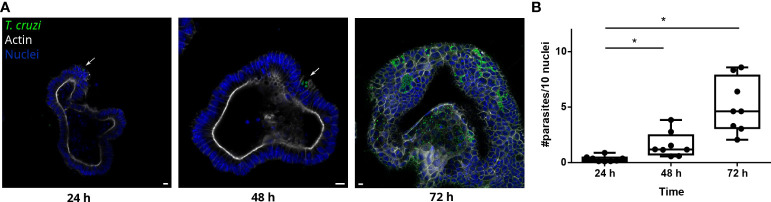
*T. cruzi* is able to invade and replicate inside organoids. **(A)** Representative images of organoids infected with parasites (MOI 1:15) and incubated for 24, 48 or 72 h. Arrows indicate infected cells. **(B)** Number of intracellular parasites every 10 cells at different time points. Data from one representative experiment out of 2 independent experiments (different organoid lines per experiment). Data are expressed as median and interquartile range and compared using Mann-Whitney test (n = 8) p<0.05. Scale bar: 10 μm. Nuclei (blue): Hoechst 33342 staining, *T. cruzi* (green): anti-trypanothione reductase immune-staining, and Actin (white): phalloidin Texas Red staining. *p<0.05.

During the chronic stage of the infection, the colonization of the intestinal tissue by *T. cruzi* is expected to occur *via* extravasation of blood circulating trypomastigotes to the interstitial space, followed by the invasion of cells from the basal layer of the organ. Smooth muscle and nervous system cells surrounding the colon have been shown to be a target of parasite infection (Vazquez et al., 2015; Ward et al., 2020; Khan et al., 2021). However, the interaction of *T. cruzi* with the intestine has been explored only by traditional cell culture techniques (**Figure 1**), and still there are no reports on the capacity of the parasite to colonize colon epithelial cells, despite this organ being a major target of infection, damage, and persistence of this pathogen. Thus, to address this question, intact colon-derived organoids were co-incubated for 2 h with *T. cruzi* trypomastigotes isolated from infected Vero cells, and cell invasion was monitored by confocal microscopy (**Figure 2A**). The representative image of **Figure 2B** shows that under this condition, only a minor fraction of colonoid cells are infected by the parasite. Suspecting that this may be caused by a physical impediment of the parasites to move freely through the matrix, next, the organoids were exposed to trypomastigotes before embedding them into the matrix. Thus, organoids were removed from the Cultrex BME matrix without disrupting them and were incubated with trypomastigotes for 2 h in suspension (MOI 1:15, epithelial cells:trypomastigotes) before replating them in BME matrix (**Figure 2A**). As shown in **Figure 2C**, this procedure led to a significant increase in the number of colonoids’ infection foci that, nonetheless, were restricted to some cells of the organoids. To further test whether this pattern of parasite colonization is specific, the intact colonoids were infected in suspension with a higher MOI (1:100; **Figure 2D**). Although this condition resulted in an overall (but not statistically significant) increase in the number of parasites per infected cell (**Figure 2E**), the infection was yet localized in specific cells/areas of the organoids (**Figure 2D**). To study parasite kinetics in infected colonoids, parasite load was quantified by confocal microscopy at 24, 48 and 72 h post infection (**Figure 3**). At time point 24 h, only one or two parasites were detected *per* infected cell (**Figure 3A**). This number increased by 6- to 19-folds after 48 h and 72 h, indicating that the pathogen was able to replicate inside the infected cells (**Figure 3B**). An important observation is that irrespective of the infection conditions, the internalized trypomastigotes were able to differentiate into replicative amastigotes, visualized as small rounded cells, which actively divided inside the colonoid cells.

### 3.2 *Trypanosoma cruzi* is able to infect proliferative and non-proliferative cells

**Figure 4:**
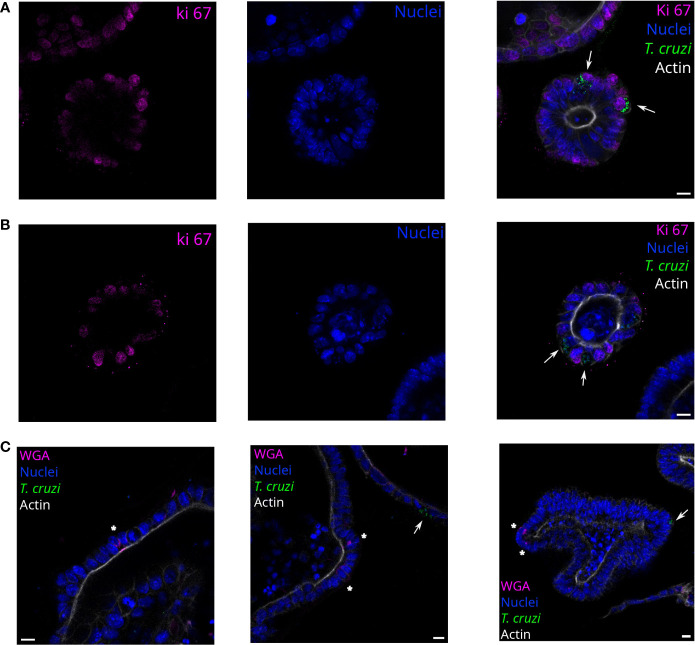
*T. cruzi* infects proliferating or non-proliferating cells but not goblet cells. Representative images of organoids infected with parasites (MOI 1:15) and incubated for 72 h. Arrows indicate infected cells. Parasites are able to infect Ki67 positive **(A)** and negative **(B)** cells. Nuclei (blue): Hoechst 33342 staining, *T. cruzi* (green): anti-mitochondrial peroxiredoxin immune-staining, and Actin (white): phalloidin Texas Red staining. **(C)** Labeled goblet cells (indicated with *) were uninfected, while some infected cells were detected in the same field. Nuclei (blue): Hoechst 33342 staining, *T. cruzi* (green): anti-mitochondrial peroxiredoxin immune-staining, Actin (magenta): phalloidin-Alexa Fluor 647 staining, and goblet cells (white): WGA-Alexa Fluor 555 staining. Scale bar: 10 μm.

Since the infection sites were restricted to some cells of the organoids regardless of the MOI used, we were interested in determining if the parasites had a preference for infecting proliferative or non-proliferative cell types. Thus, parasites and organoids were incubated for 2 h in suspension with a 1:15 MOI and then further cultured for 72 h. The organoid-derived proliferating cells (stem cells and progenitor cells) were detected using anti-Ki67 staining (**Figure 4**). Both proliferative (Ki67 positive; **Figure 4A**) and non-proliferative cells (Ki67 negative; **Figure 4B**) were infected by parasites. On the other hand, specialized secretory cells such as goblet cells were identified by WGA staining of the organoids but, interestingly, no parasites were infecting them (**Figure 4C**).

### 3.3 *Trypanosoma cruzi* invades colon-derived cells from their apical side in a discrete fashion

**Figure 5:**
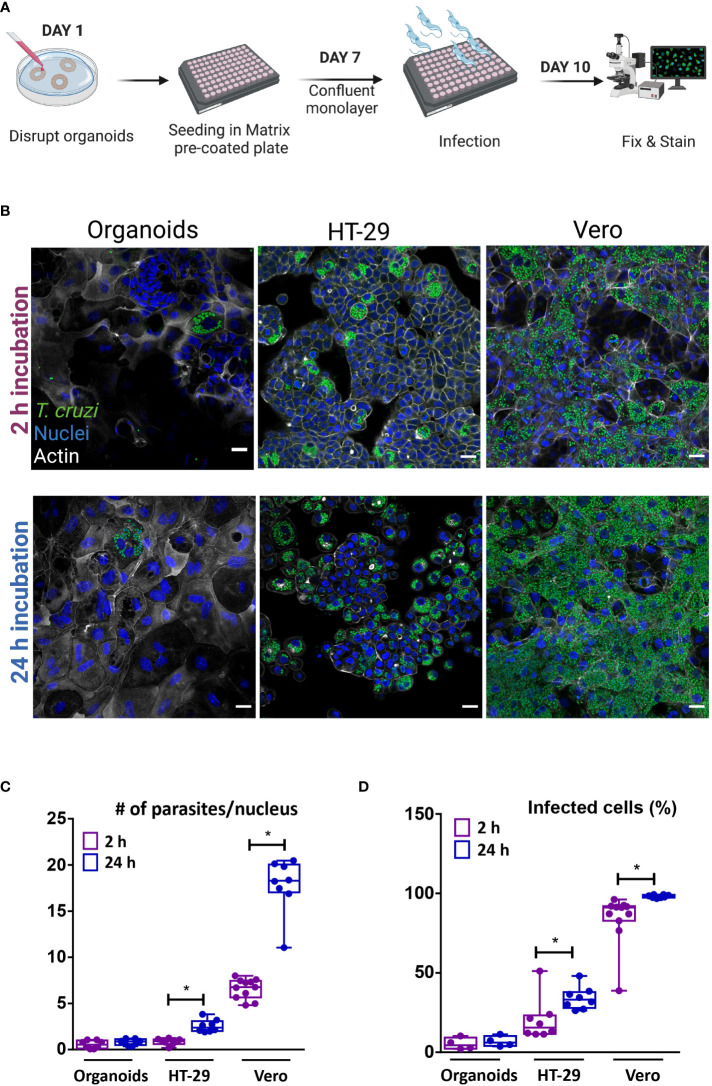
Apical organoid and cell-line infection by *T cruzi*. **(A)** Schematic representation of the infection protocol. Vero, HT-29 cells and murine colon-organoid derived cells were seeded as monolayers. After reaching confluence, cells were incubated with the parasites for 2 h or 24 h at 37°C and further incubated at 37°C for 72 h. Created with BioRender (agreement number WV2506JDUP) **(B)** Representative images of 2D infected cells (MOI 1:15). **(C)** Number of intracellular parasites per host-cell nucleus over a 72 h period of infection. **(D)** Quantification of the percentage of cells infected with at least one parasite. Infection rate was improved with longer incubation time for HT-29 and Vero cells, while organoid-derived monolayers showed similar parasite load, regardless of time exposure. Data from one representative experiment out of 2 independent experiments (different organoid lines per experiment). Data are expressed as median and interquartile range and compared using Mann-Whitney test (n = 8), *p<0.05. Scale bar: 25 µm. Nuclei (blue): methyl green staining, *T. cruzi* (green): anti-trypanothione reductase immune-staining, and Actin (white): phalloidin Texas Red staining.

In order to facilitate the apical access of the parasite to the full repertoire of epithelial cells present in the colon-derived organoids, organoid monolayers were prepared and used for infection. To generate a simplified monolayer culture system similar to the conventional 2D culture, the organoids were removed from the BME matrix, dissociated both mechanically and enzymatically, and the cell suspension was seeded in a pre-coated multi-well plate (**Figure 5A**). Once the monolayer reached confluence, trypomastigotes were added at a 1:15 MOI (epithelial cells:trypomastigotes) and incubated for 2 or 24 h. Upon removal of non-internalized parasites, the incubation was extended for a total of 72 h (**Figure 5B**). For comparison purposes, tumoral cell lines from human colon (HT-29 cell line) and green monkey kidney (Vero cell line) were infected with *T. cruzi* under the same conditions used for organoid cells. Similar to the results obtained in the basolateral infections of intact organoids, *T. cruzi* infected only a discrete number of epithelial cells despite the large area of cells available in the 2D culture (**Figure 5B**). Extending the time the monolayer was exposed to extracellular trypomastigotes (i.e. from 2 to 24 h) did not result in significant changes in the number of infected cells or parasite burden per cell (**Figure 5C, D**). Notably, a short infection time (2 h) also led to a selective infection of human intestinal epithelial cells HT-29, though the number of parasites/cells and of infected cells was slightly higher than that observed in organoid-derived cells. Contrary to the results obtained for the organoid monolayer, extending the exposure of the HT-29 intestinal cell line to trypomastigotes (24 h incubation) resulted in a significant 3- to 4-folds increase in the level of infected cells and parasites/cell (**Figure 5C, D**). Nonetheless, the level of infection achieved on this cell line was not as massive as that obtained in Vero cells (**Figure 5B**). In fact, a 2 h incubation of this cell type with trypomastigotes resulted in 75% infection of the cell monolayer, a value that reached a maximum of 98% when the incubation was extended to 24 h (**Figures 5B, D**). The number of amastigotes per Vero cell doubled when the incubation time was extended from 2 to 24 h, which can likely be ascribed to a higher number of trypomastigotes invading a single cell (**Figures 5C**). For all conditions tested, the parasite was able to complete its differentiation and multiplication cycle inside the different host cells. Overall, this data indicates that certain organoid- and tumor-derived cells from the colon are susceptible to infection by *T. cruzi* Dm28c, while stable cell lines from primate-derived kidney are highly permissive to parasite invasion.

## 4 Discussion

Intestinal organoids have been used in the study of the biology and development of the intestinal epithelium (Clevers, 2016; Puschhof et al., 2021), as well as in understanding the interaction with different pathogens and the gut microbiota (Han et al., 2021). However, these models had yet not been applied to model CD (Breyner et al., 2020). The intestinal-related infection, particularly in the colon segment, during the chronic stage of CD, plays an important role as a parasite reservoir that is refractory to drug treatment and immunological recognition. The lack of appropriate models to study this parasite-organ interaction in depth has prompted us to undertake the development and preliminary characterization of infection protocols of colon-derived organoids, which are closer to resembling physiological conditions.

The conditions for infecting murine colon-derived organoids in 3D and 2D formats with *T. cruzi* trypomastigotes are described here, to our knowledge, for the first time. The method for the infection of mouse colon-derived organoid epithelial sheets here reported is simple and economic. It is adapted to a 96-well plate format, where no transwell is needed and media and staining solutions volume are significantly reduced when compared to other chamber or plate formats. Performing the infection of intact organoids embedded in an extracellular matrix, or devoid of the supportive matrix with culture-derived (highly infective) trypomastigotes proved successful. Mimicking tissue colonization in the chronic stage of CD (i.e. basolateral infection), the parasites were capable of invading and replicating in epithelial cells. Infection experiments at extended time points may reveal if the parasites are also able to reach the organoid lumen by paracellular or intracellular migration. Notably, the invasion appears to be cell-type specific because even exposing the host cells to a 100-fold excess of parasite cells, yet resulted in a discrete number of colonoid cells being permissive to infection. A similar outcome was obtained when the infection was performed from the apical side of a 2D monolayer of organoid cells. Although the primary site of body penetration during oral transmission of CD is yet unknown, our result indicates that the parasite is able to establish infections in colon epithelial cells when accessing them from the lumen. Therefore, the 2D culture system may be of interest to studying the oral transmission route of *T. cruzi* since it avoids the use of microinjection techniques to access the organoid from the luminal surface.

An intriguing finding was that discrete foci of infected cells were observed in both spatial arrangements of the murine organoids, 3D and 2D, as well as in human-derived colon cell lines. This differed markedly from the massive infection achieved in a kidney-derived epithelial cell line of primate origin. Our results suggest that the reason for the susceptibility of certain colon-derived cells to be infected by *T. cruzi* cannot be ascribed to species-specific origin of the host cells nor to cell polarization because, as mentioned above, the phenomenon was observed in murine- and human-derived cells, the former infected from both the apical and basolateral side. It is therefore tempting to speculate that a combination of host (e.g., receptors, different cell types) and pathogen-specific factors and signals determine this selectivity. In fact, *T. cruzi* entry to the host cells is a multifactorial process that may follow actin-dependent or lysosome-dependent and -independent mechanisms (de Souza et al., 2010). Although not yet fully understood, the current evidence supports that for invading non-phagocytic cells, *T. cruzi* exploits a highly conserved cellular pathway for the repair of plasma membrane lesions (Fernandes and Andrews, 2012). This process is largely determined by parasite-induced signaling pathways that involve primary membrane injury, followed by lysosome recruitment and content release at the site of entry, which favor an active internalization of the pathogen inside ceramide/lysosome enriched vacuoles. Such a peculiar mechanism of pathogen entry along with the fact that the membrane of muscle cells is frequently exposed to mechanical stress, and, therefore, has an exacerbated turnover for membrane repair, has been a major argument to explain the tropism of the parasite for muscle cells. On the other hand, non-professional phagocytic cells metabolically- (starvation) or pharmacologically-induced to undergo autophagy proved highly susceptible to *T. cruzi* infection (Romano et al., 2009). Also, the composition of the membrane (microdomains) has been demonstrated to be an additional factor contributing to *T. cruzi* invasion of phagocytic and non-phagocytic cells (Barrias et al., 2007; Fernandes et al., 2007). Which of these or, perhaps, novel factors contribute to the observed tropism of *T. cruzi* for certain colon-derived cells needs to be further investigated. In our experiments, proliferative and non-proliferative cells were infected by parasites, but specific cell types were not yet identified to be responsible for the discrete zones of infection within the organoid. However, we did not observe mucin-secreting cells (goblet cells) infected by *T. cruzi*. In the intestine (and also other tissues), goblet cells are specialized in creating a protective mucus layer that mechanically prevents the contact of epithelial cells with external factors (from microorganisms to macromolecules; Birchenough et al., 2015). Thus, it is tempting to speculate that this dense barrier of heavily glycosylated proteins is responsible for blocking the access of the parasite to the goblet and surrounding cells. At this point is worth recalling that our experimental conditions for growing organoids promote stem cell proliferation rather than cell differentiation. Therefore, further experiments using differentiated organoids should be performed to better model the interactions of *T. cruzi* with the intestinal epithelia.

Generally compared to the *in vivo* methods, intestinal organoids can provide a less expensive and more rapid model to study intestinal cell-*T. cruzi* interaction. Since the availability of tissue from patients with digestive forms of CD is scarce, the murine intestinal organoids are an alternative to bypass this limitation (Breyner et al., 2020). However, future studies in human organoids derived from healthy donors may help improve the infection model to better mimic human disease. Moreover, while murine intestinal organoids, such as those used in this study, present only the epithelial compartment, organoid co-culture systems with other cell types such as nervous, stromal (Pastuła et al., 2015), and immune cells (Noel et al., 2017) have been developed and may be very useful to increase the complexity of the system for dissecting host-pathogen interaction in CD.

## 5 Conclusion

A simple and economic method that allows to model the infection of 2D and 3D intestinal organoids from murine colon by *T. cruzi* has been established. The study delivered interesting preliminary information about parasite invasion and proliferation in the host tissue. The organoid model represents a valuable tool to understand several aspects of the host-pathogen interaction that may translate into more effective treatments of CD.

## Data availability statement

The raw data supporting the conclusions of this article will be made available by the authors, without undue reservation.

## Ethics statement

The animal study was reviewed and approved by Comisión de Ética en el Uso de Animales (CEUA) - Institut Pasteur de Montevideo (Protocol #002-21).

## Author contributions

HD contributed to experimental design, the data acquisition, analysis and writing of the manuscript. RP contributed to the experimental design, data acquisition and analysis. CQ contributed to data acquisition. AM contributed to data acquisition and to experimental design. MB-F contributed to funding acquisition and experimental design. MAC contributed to experimental design. All authors contributed to the article and approved the submitted version.
